# Bilateral Lumbosacral Plexopathy As the Initial Manifestation of Systemic Sarcoidosis: A Case Report

**DOI:** 10.7759/cureus.54086

**Published:** 2024-02-12

**Authors:** Andrés F Cardona-Cardona, Sehreen Mumtaz, Lisa Balistreri, Rupert Stanbourough, Ronald Butendieck, Benjamin Wang, Andy Abril, Majithia Vikas, Florentina Berianu

**Affiliations:** 1 Rheumatology, Universidad de Antioquia, Medellín, COL; 2 Rheumatology, Mayo Clinic Florida, Jacksonville, USA; 3 Radiology, Mayo Clinic Florida, Jacksonville, USA

**Keywords:** radiculopathy, neuropathy peripheral, lumbosacral plexus, peripheral nervous system diseases, neurosarcoidosis, sarcoidosis

## Abstract

Neurosarcoidosis is one of the most relevant involvements in systemic sarcoidosis and can be the initial presentation. Its diagnosis is often considered difficult because of unusual clinical manifestations or diagnostic mimics. The peripheral nervous system is less frequently involved than the central nervous system, although it may also lead to irreversible neurologic impairment. Lumbosacral plexopathy in sarcoidosis is a rare presentation and has been scarcely described in anecdotal case reports and small case series.

We describe the case of a 61-year-old female who presented with right inguinal pain, right thigh weakness, and gait limitation, with imaging evidence of bilateral lumbosacral plexopathy as the initial manifestation of systemic sarcoidosis and subsequently developed joint and pulmonary involvement.

This case report aims to bring awareness of this involvement as a possible initial manifestation of systemic sarcoidosis and mention key features of the differential diagnosis. Prompt recognition and treatment may prevent neurologic impairment.

## Introduction

Sarcoidosis is a multisystem granulomatous inflammatory disease of unknown etiology and may affect various organs with myriad clinical manifestations [1]. It is estimated to affect the neurological system in 5-15% of cases, usually occurring within the first two years of illness, and neurological disease indicates systemic involvement with uncontrolled inflammation [2,3]. Neurosarcoidosis can affect meninges, brain parenchyma, spinal cord, cranial nerves, peripheral nerves, and muscles [3]. Peripheral nerve involvement includes granulomatous neuropathy or small fiber non-granulomatous neuropathy. The former can occur as polyneuropathy, polyradiculopathy, mononeuropathy, mononeuritis multiplex, or lumbosacral plexopathy [4]. The diagnosis of neurosarcoidosis is established by the clinical and imaging manifestations, histopathological findings, and exclusion of other causes [2]. Even though typical presentations of sarcoidosis allow prompt diagnosis, in a substantial proportion of cases, the diagnosis is often difficult because of unusual clinical manifestations [5]. Herein, we report a case of systemic sarcoidosis with bilateral lumbar plexopathy as the initial manifestation, a significant involvement scarcely described in the medical literature. Written informed consent for publication was obtained from the patient. This report and its discussion highlight the clinical, electrodiagnostic studies and imaging features to recognize this rare manifestation, explore the differential diagnosis, and provide some recommended treatments. 

## Case presentation

A 61-year-old Caucasian female patient presented to our rheumatology outpatient clinic with a seven-year history of right inguinal pain radiating to the right anterior thigh, resulting in gait limitation. One year prior to the presentation, she noted right thigh weakness, and four months before the presentation, she developed inflammatory pain in her right hand without swelling. The patient had a history of hyperthyroidism, asthma, and right hip arthroplasty. Initially, the hip pain was thought to be related to her previous hip surgery. Nevertheless, a right hip magnetic resonance imaging (MRI) from another institution did not reveal alteration related to the arthroplasty but showed right lumbar plexopathy. Hence, she was referred to rheumatology and neurology.

The review of symptoms was positive for fatigue and diffuse alopecia. The patient denied any weight loss, fever, enlarged or painful lymph nodes, red or painful eyes, skin rash, sicca symptoms, mouth ulcers, chest pain, shortness of breath, or recent viral infections. The physical exam was notable for pain upon palpating the second to fifth metacarpophalangeal joints, but there was no synovitis, sclerodactyly, or dactylitis. There was no pain in her hips with passive hip movement, but she had mild thigh atrophy prominently on the right side. Hip flexor strength was reduced, 3/5 on the right and 4/5 on the left. Sensation to pinprick was normal, but she had moderately decreased vibratory sensibility involving her right leg. The remainder of the physical examination was unremarkable.

Laboratory testing revealed a normal cell blood count; her C-reactive protein was 4.2 mg/L (reference range (RR): <5.0 mg/L), erythrocyte sedimentation rate was 9 mm/1st hour (RR: 1-20 mm/1st hour), and creatinine was 0.75 mg/dL (RR: 0.59-1.04 mg/dL). Rheumatoid factor, cyclic citrullinated peptide antibodies, anti-nuclear antibodies, anti-extractable nuclear antigens antibodies, anti-neutrophil cytoplasmic antibodies, HLA-B27, and HIV test were negative. Other laboratory tests were unremarkable, including a normal cerebrospinal fluid analysis, blood glucose, hemoglobin A1C, serum electrophoresis, and urinalysis. Additionally, previous external electromyography (EMG) with nerve conduction studies reported reduced compound muscle action potential (CMAP) amplitudes in fibular muscles without other findings.

**Figure 1:**
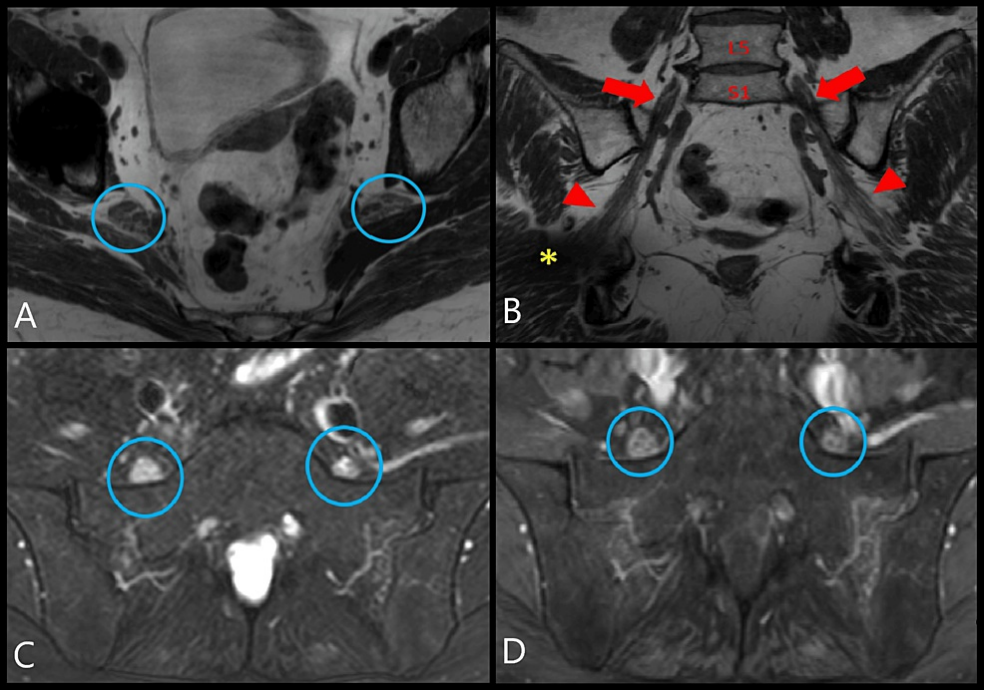
MRI of the lumbosacral plexus. PD 3D isovolumetric MRI of the lumbosacral plexus in the oblique axial (A) and oblique coronal (B) planes showed marked diffuse enlargement of the bilateral sciatic nerves (blue circles) beginning at the level of nerve roots (red arrows indicate the L5 nerve roots) extending distal through the sciatic notch (red arrowheads). There was a metal artifact from a right hip arthroplasty (yellow asterisk). Axial T2 SPAIR MRI (C) and axial T1 fat-saturated post-contrast MRI (D) of the lumbosacral plexus at the level of the L5 nerve roots (blue circles) contributing to the lumbosacral plexus proximally. Image C evidenced enlargement and abnormal hyperintense (bright) signal of the L5 nerve roots. Image D exhibited perineural enhancement surrounding the individual L5 nerve fascicles implying active inflammation. MRI, magnetic resonance imaging; PD, proton density; SPAIR, spectral attenuated inversion recovery

**Figure 2:**
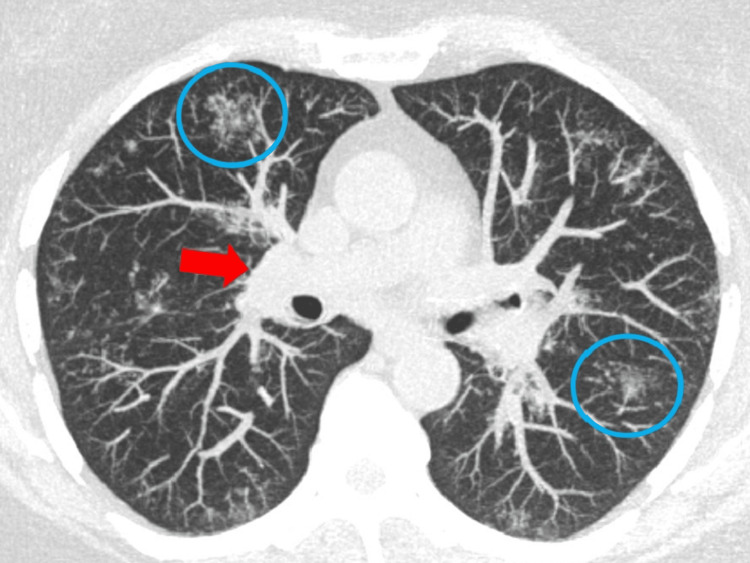
Chest CT image. Axial MIP non-contrast chest CT image showed perilymphatic micronodules in both lungs (larger clusters demarcated with blue circles) and hilar lymphadenopathy (red arrow). These findings are consistent with sarcoidosis. MIP, maximum intensity projection; CT, computed tomography

**Figure 3:**
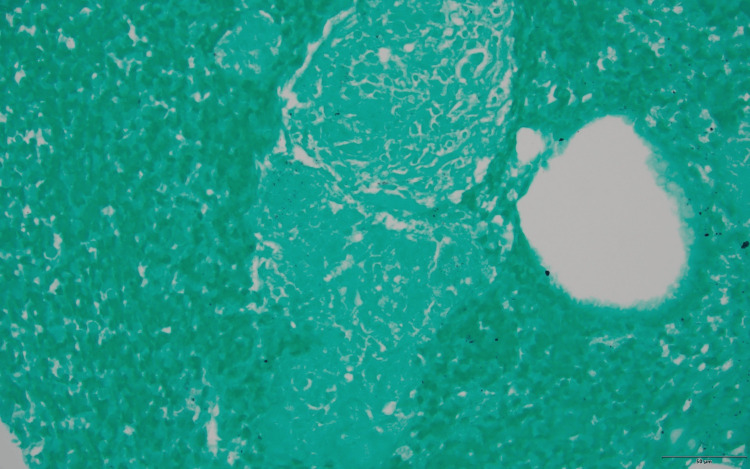
Grocott–Gömöri's methenamine silver stained section of the transbronchial lymph node biopsy. High power magnification showed no evidence of fungal yeasts or hyphal elements (40x).

**Figure 4:**
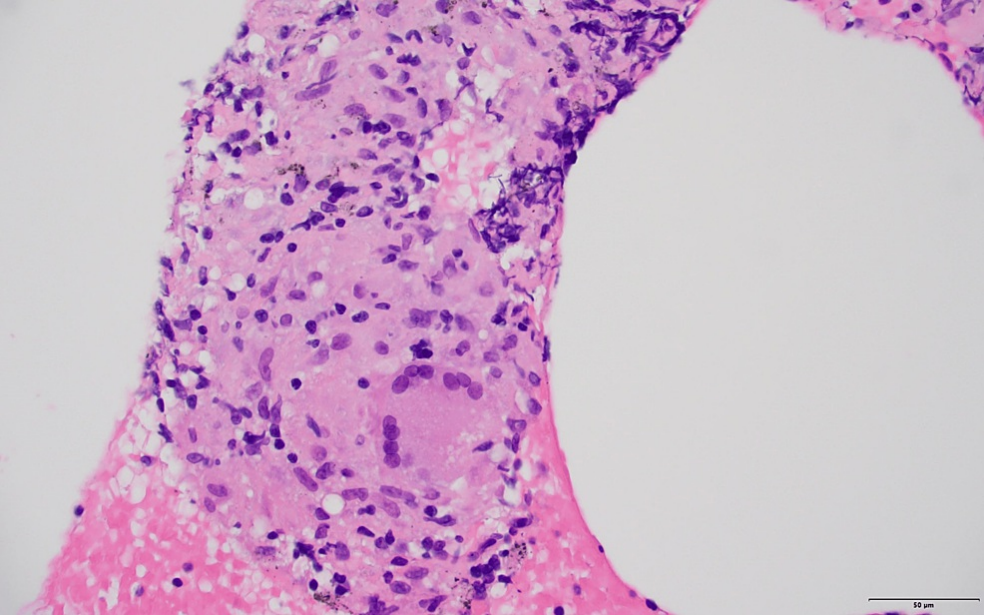
Hematoxylin and eosin stained section of the transbronchial lymph node biopsy. High power magnification showing multinucleated giant cells and histiocytic aggregates consistent with non-necrotizing epithelioid granulomas (40x).

An MRI of the left hand demonstrated carpal and metacarpophalangeal joint synovitis and extensor tenosynovitis. Lumbar plexus MRI with and without contrast revealed marked diffuse enlargement with abnormal signal and enhancement of the lumbosacral plexus, proximal sciatic, and femoral nerves, more significant on the right side, and mild diffuse muscle atrophy surrounding hip girdles and thighs (Figure 1). There was no mass or focal nodular signal abnormality to suggest a neoplastic or infectious etiology. Chest radiographs showed left perihilar nodularity and mild bibasilar reticulations. The subsequent chest computed tomography (CT) demonstrated multiple scattered perilymphatic micronodules in both lungs and mildly enlarged mediastinal and bilateral hilar lymph nodes (Figure 2). Endobronchial ultrasound with transbronchial lymph node biopsy noted noncaseating granulomas, negative cultures, and unremarkable flow cytometry (Figure 3 and Figure 4).

**Figure 5:**
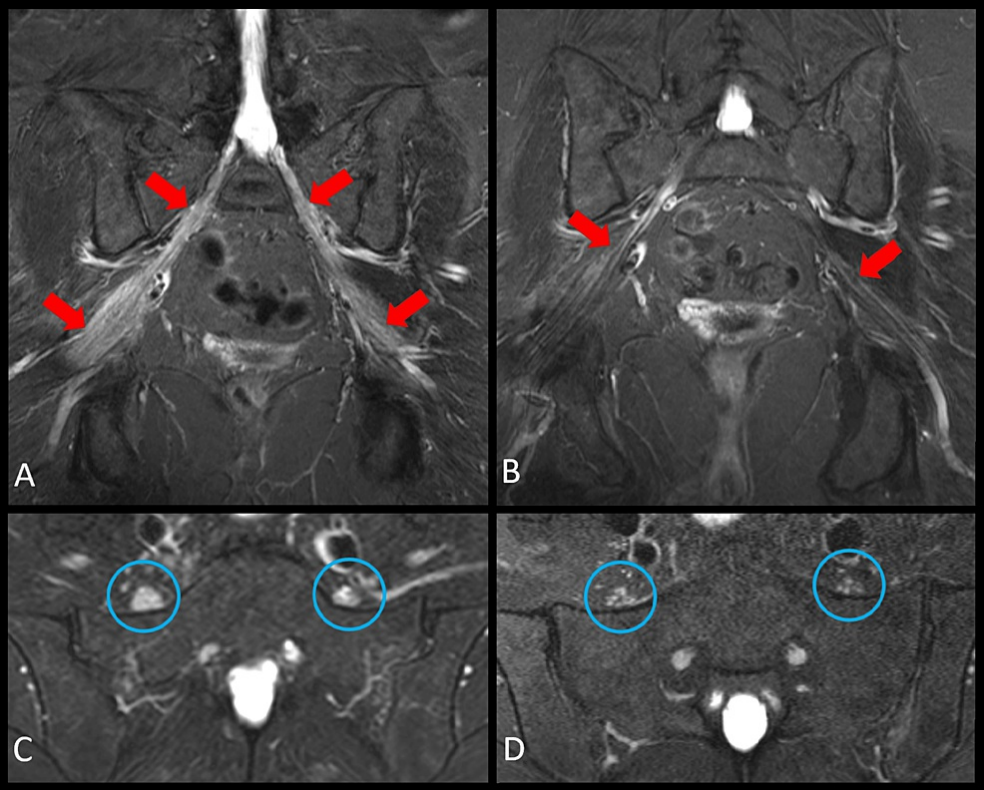
Pre- and post-treatment MRI comparison. Coronal T2 STIR MRI of the lumbosacral plexus prior to diagnosis (A) and post-treatment (B). Image A showed marked diffuse enlargement and hyperintense (bright) signal of the bilateral sciatic nerves (red arrows). Image B evidenced the near-normal reduction of sciatic nerve caliber and signal post-treatment. Axial T2 SPAIR MRI prior to diagnosis (C) and post-treatment (D) at the level of the L5 nerve roots (blue circles). Image C presented enlargement and abnormal hyperintense (bright) signal of the L5 nerve roots. Image D exhibited a near-normal reduction of nerve caliber and signal post-treatment. MRI, magnetic resonance imaging; STIR, short-tau inversion recovery; SPAIR, spectral attenuated inversion recovery

Considering the compatible clinical course, bilateral lumbar plexopathy, joint and lung involvement, and positive biopsy findings, a diagnosis of systemic sarcoidosis was made. The patient received treatment with oral prednisone 10 mg daily and oral methotrexate 15 mg weekly for six months without improvement. Hence, intravenous infliximab was added at a dose of 5 mg/kg with a loading regimen at weeks zero, two, and six, and then every eight weeks. A year after the diagnosis, her fatigue, joint symptoms, and right inguinal region pain resolved, but the weakness persisted and felt to represent a neurological sequela. However, a follow-up MRI of the lumbar plexus presented improvement with a reduction of sciatic nerve caliber and signal post-treatment (Figure 5). She did not develop any respiratory manifestations, pulmonary function tests were normal, and a new chest CT showed a decrease in the size of the mediastinal lymph nodes and significant improvement in the perilymphatic nodularity.

## Discussion

Neurosarcoidosis is the fifth most frequent involvement in sarcoidosis after pulmonary, ocular, dermatological, and lymph node involvement [6]. It affects women more than men, with a peak incidence in the third to fifth decade [4]. Neurologic symptoms can be the initial feature in up to 52% of the cases, may have an acute or chronic onset, and can present with a monophasic, remitting-relapsing, or chronically progressive course [1,4].

Peripheral nerve involvement occurs in 2-26% of patients with systemic sarcoidosis [1]. It is classified as non-granulomatous small fiber neuropathy and granulomatous neuropathy. The latter may manifest as cranial nerve involvement, distal symmetric sensorimotor polyneuropathy, asymmetric polyradiculoneuropathy, pure sensory or motor neuropathy, mononeuritis multiplex, polyradiculopathy, mononeuropathy, lumbosacral plexopathy, chronic inflammatory demyelinating polyneuropathy, and multifocal motor neuropathy with conduction block [4]. Granulomatous inflammatory infiltration of the nerves, vasculitis, or necrotizing vasculitic changes seem to be the mechanisms of neuropathic involvement in sarcoidosis, based on findings derived chiefly from studies of large fiber neuropathies [5,7].

Lumbosacral plexopathy in sarcoidosis is uncommon, but many cases are probably unrecognized. It has been documented in anecdotal case reports and small case series [8-10]. Tulloch et al. described a 51-year-old female with systemic sarcoidosis who developed contralateral lumbosacral plexopathy following lumbar microdiscectomy and responded to high-dose corticosteroids [8]. Zuniga et al. found in their case series of 10 cases of systemic sarcoidosis a 63-year-old male patient with pulmonary sarcoidosis, left lumbosacral plexopathy, and axonal sensorimotor neuropathy [9]. Burns et al. analyzed 57 cases from the Mayo Clinic with sarcoidosis neuropathy based on their electrodiagnostic and MRI features [10]. They found six cases with multiple mononeuropathies, 22 polyradiculoneuropathies, nine polyradiculopathies, 19 polyneuropathies, and just one lumbosacral radiculoplexus neuropathy. Neuropathic symptoms predated sarcoidosis diagnosis in 65% of the cases. The authors inferred a focal or multifocal pathologic process involving most classes of nerve fibers [10].

The clinical picture of lumbosacral plexopathy depends on what portion of the plexus is involved [11]. Plexopathies are characterized clinically by progressive numbness, pain, and paresthesia, followed by weakness that is not in the distribution of a single nerve or nerve root [11,12]. Lumbar plexopathies typically manifest pain and sensory loss localized to the anterolateral and medial thigh region and weakness involves the quadriceps, hip flexors, and hip adductors, similar to the patient in this case report [13]. In sacral plexopathies sensory loss and weakness involve the posterior thigh, the leg, and the foot [13]. In sarcoidosis, the symptoms of lumbosacral plexopathy have been described as pain overshadowing asymmetric weakness or sensory loss involving proximal nerve segments [10,11]. These symptoms can be the only manifestation upon initial evaluation, and diagnostic markers for neurosarcoidosis are lacking. Therefore, their underlying diagnosis may be overlooked.

Electrodiagnostic studies can assist in localizing the disorder to the lumbosacral plexus. Plexopathies may occur along with root and peripheral nerve involvement, causing radiculoplexus neuropathies [11]. Typical findings are low CMAP amplitudes in muscles innervated by the involved nerve segments within the plexus and conduction velocities are usually normal or mildly reduced [13-15]. However, differentiation is not easy, many of the sensory nerves that arise from the lumbosacral plexus do not have standardized nerve conduction tests, and some cases are technically challenging [12,13,15].

MRI is the imaging study of choice to evaluate the lumbosacral plexus [13,16]. Abnormal MRI findings in lumbosacral plexopathies include enlargement or edema of nerve segments, increased T2 signal intensity, and an abnormal focal or diffuse contrast enhancement on fat-saturated T1-weighted images [13,16]. Still, there are no characteristic features of sarcoidosis. A sensory nerve biopsy (often from the sciatic nerve) taken from the site of the imaging abnormality can help diagnose lumbosacral plexopathies [11]. Nevertheless, a biopsy of a relatively safe and accessible nonneural site is recommended to establish a pathologic diagnosis [5].

Many disorders can involve the lumbosacral plexus. Table 1 summarizes the differential diagnosis with some key features. The lumbosacral plexus is a deep structure in the pelvis, and it is not as susceptible to direct trauma as the brachial plexus, although it could be associated with sacral fractures or sacroiliac joint dislocations [11,13,14]. Postoperative lumbosacral plexopathy can result from orthopedic procedures such as total hip arthroplasty [8,11]. In this case report, the hip arthroplasty was done after the onset of the symptoms. Additionally, the bilateral involvement favors sarcoidosis as the likely diagnosis.

**Table 1:** Differential diagnosis of lumbosacral plexopathy. DILS, diffuse infiltrative lymphocytosis syndrome; HIV, human immunodeficiency virus infection; LSRPN, lumbosacral radiculoplexus neuropathy

Etiology	Mechanism and key features
Trauma [13,14]	High-energy trauma or gunshot wound.
	Sacral fractures or sacroiliac joint dislocations.
	Intrapartum lumbosacral plexopathy.
Surgery [11,13]	Direct trauma associated with surgical or anesthetic procedures (total hip arthroplasty).
	Postsurgical inflammatory neuropathy (postoperative onset and continued progressing).
Inflammatory	
Type 2 diabetes [11,17]	Diabetic LSRPN.
	Previous medical history of diabetes but could be well-controlled at presentation.
	Monophasic illness that worsens for about six months and then gradually improves.
	Autonomic features (orthostatic symptoms, constipation, urinary or sexual dysfunction).
Non-diabetic LSRPN [11]	Middle age and older, with an average age of about 65 years.
	Typically is associated with significant weight loss.
Sarcoidosis [10,11]	Sensory predominant neuropathy with pain overshadowing weakness or sensory loss.
	Usually is asymmetric and involves proximal nerve segments.
	Can be the initial manifestation.
Vascular lesions [11,13]	Retroperitoneal hematoma, iliac artery, or abdominal aortic aneurysm (compression).
	Related to trauma, hemophilia, anticoagulant use, or hematologic malignancy.
	Acute onset of unilateral pain in the lower back or flank followed by motor weakness.
Infection [11]	Local infection (psoas abscess, Pott disease, gastrointestinal or urinary tract infections).
	Perirectal abscess in HIV patients.
	DILS in HIV patients (painful, paralytic lower limb neuropathy).
Malignancy [11]	Usually presents with the subacute onset and involves the lumbar and sacral plexus.
	Direct malignant invasion from adjacent organs (colon, cervix, and urinary bladder).
	Metastatic deposits (lymphoma, breast, or lung cancer).
	Perineural spread (prostate cancer).
	Primary nerve sheath tumors (neurofibroma, perineurioma).
	Amyloid neuropathy (focal amyloid deposition known as amyloidoma).
Radiation [11]	Radiation therapy of neoplasm (cervical, ovarian, prostate, testicular, or colon).
	It begins months or years following the radiation exposure.
	Myokymic discharges are found in approximately 60%.

Patients with diabetic lumbosacral radiculoplexus neuropathy experience additional autonomic features [13,17]. The presence of other features, such as enlarged inguinal lymph nodes, tumors, or a pulsatile femoral mass, may suggest neoplastic invasion or aneurysm as the probable underlying disease etiology [11,13]. Furthermore, radiation for malignancies in the pelvic or lower abdominal regions may involve lumbar and sacral plexus [11,13]. All these features were absent in the case presented herein, the laboratory testing ruled out diabetes and infectious disease, and the MRI did not show fractures, mass, or focal nodular signal abnormality to suggest a neoplastic or infectious etiology.

Among rheumatologic diseases, another differential diagnosis of peripheral neuropathy is Sjögren’s syndrome. The spectrum of peripheral neurologic involvement in Sjögren’s syndrome includes small fiber neuropathy, sensory polyneuropathy, sensory ataxic neuropathy (ganglionopathy), axonal sensorimotor polyneuropathy, demyelinating polyradiculoneuropathy, autonomic neuropathy, mononeuropathies or mononeuropathy multiplex, and cranial neuropathies [18]. Plexopathy has not been described in previous cases series of Sjögren’s syndrome, and this disease was discarded in this case report because of the absence of sicca symptoms, anti-nuclear antibodies, and anti-extractable nuclear antigens antibodies [19].

The Neurosarcoidosis Consortium Consensus Group has developed consensus criteria for diagnosing neurosarcoidosis involving the central and peripheral nervous system, emphasizing the need to obtain histologic confirmation of systemic sarcoidosis [5]. Although lumbosacral plexopathy is an uncommon presentation, according to these criteria, the case reported has a diagnosis of probable peripheral nervous system neurosarcoidosis given the clinical presentation, MRI findings, pathologic confirmation of systemic granulomatous disease consistent with sarcoidosis in the lymph nodes, and rigorous exclusion of other causes.

Although spontaneous improvement or remission occurs in about 60% of patients with neurosarcoidosis, the mortality rate in all forms of sarcoidosis is from 1 to 5% due to severe pulmonary, cardiac, or neurologic disease [3]. Because of the rarity of plexopathy in sarcoidosis, there are no randomized controlled trials, and its treatment is based on regimens used in peripheral neuropathy. Treatment includes immunosuppressive therapy, pain management, and physical therapy [11]. The first line of treatment is high-dose corticosteroids. Methotrexate, azathioprine, and mycophenolate mofetil are used as steroid-sparing agents, and TNF-α antagonists or cyclophosphamide may be considered in refractory cases [1,4].

## Conclusions

This case report brings awareness of lumbosacral plexopathy as a possible initial manifestation of systemic sarcoidosis. It is a rare presentation and has been scarcely described in anecdotal case reports and small case series, although it may generate irreversible neurologic impairment, like in the present report. The clinical and imaging features allow the recognition of this involvement, but high suspicion is needed to ensure prompt recognition and treatment. Diagnostic mimics should be considered, and their key features are helpful for the differential diagnosis. This report also highlights the relevance of another organ system involvement and pathologic proof in other tissues to ensure the diagnosis of sarcoidosis due to the significant challenge regarding the difficulty of obtaining neural tissue for histologic analyses.
